# The posterior femoral cartilage can be used as an anatomical reference for the creation of the femoral tunnel in anterior cruciate ligament reconstruction

**DOI:** 10.1002/jeo2.70403

**Published:** 2025-09-05

**Authors:** Viktor Nelson Mazzola Corrêa, Nayra Deise dos Anjos Rabelo, Alfredo dos Santos Netto, Victor Marques de Oliveira, Luiz Gabriel Betni Guglielmetti, Ricardo de Paula Leite Cury

**Affiliations:** ^1^ Department of Orthopedics and Traumatology Knee Surgery Group, School of Medical Sciences, Santa Casa of Sao Paulo Sao Paulo Sao Paulo Brazil

**Keywords:** arthroscopy, anterior cruciate ligament, anterior cruciate ligament reconstruction, femoral tunnel

## Abstract

**Purpose:**

The objective of the study was to evaluate the accuracy of femoral tunnel positioning in the reconstruction of the anteromedial (AM) bundle of the Anterior Cruciate Ligament (ACL) using the most proximal and posterior portion of the lateral femoral condyle cartilage (Point C).

**Methods:**

From December 2022 to December 2023, 47 patients underwent anterior cruciate ligament reconstruction (ACLR) in outside in manner using Point C as an anatomical landmark for AM bundle ACLR. After the procedure, the patients underwent tomographic evaluation to assess the accuracy of the positioning, using Bernard's quadrants. Two evaluators at three different times measured the percentages for each case.

**Results:**

The average distance from Point C to the anterior portion of the lateral femoral condyle was 23.3 mm, and the average correlation value of Point C with the centre of the AM bundle in the horizontal coordinate intraoperatively was 7.7 mm. The average depth values (X coordinate) for evaluator 1 at Time 1 were 23.6%, and at Time 2 were 23.6%. The average height values (Y coordinate) at Time 1 were 22.7%, and at Time 2 were 22.1%. The analysis by the second evaluator at the third time point had an average X coordinate of 23.6% and Y coordinate of 22.3%.

**Conclusion:**

Point C proved to be a reliable anatomical landmark, exhibiting good accuracy in mimicking the AM bundle in the femoral tunnel during ACLR.

**Level of Evidence:**

Level IV.

**Abbreviations**
ACL: anterior cruciate ligament
ACLR: anterior cruciate ligament reconstruction
AM: anteromedial
BMI: body mass index
CT: computed tomography
ICC: intraclass correlation coefficient
IKDC: International Knee Documentation Committee
mm: millimetres
SEE: standard error of estimate
SEM: standard error of measurement

## INTRODUCTION

The anatomical positioning of the tibial and femoral tunnels in anterior cruciate ligament reconstruction (ACLR) has been shown to provide better restoration of anterior tibial translation, rotatory knee stability, and knee kinematics [1], leading to improved clinical and biomechanical outcomes [4]. Despite the good results of ACLR, some studies have shown a revision rate up to 25% [10]. Multifactorial etiologies, including technical, traumatic, and biological factors, represent the primary causes of revision ACLR [32]. Furthermore, evidence suggests that 47.6% of patients requiring revision ACLR have a non‐anatomical femoral tunnel [20], emphasising the continued debate over the most reliable methods for achieving anatomical tunnel positioning [1]. An analysis with 221 high‐volume surgeons evaluating the accuracy of femoral tunnel creation showed that 18% of surgeons placed the femoral tunnel outside the native region of the anterior cruciate ligament (ACL), even though they intended to place it anatomically [25]. This is due to the difficulty in identifying the location of the femoral footprint during ACLR.

The femoral footprint of the ACL has been measured by Bernard et al. [2] using the Bernard and Hertel quadrant method, a radiographic technique based on a 4 × 4 grid applied to lateral knee radiographs. In this method, the ACL femoral insertion is located at 25% of the total sagittal diameter of the lateral femoral condyle along Blumensaat's line (deep to shallow), and 29% of the condylar height measured perpendicularly from Blumensaat's line (high to low). However, other studies have reported different percentages of the ACL femoral footprint [33], and the method has been adapted for evaluation using computed tomography [14].

The concept of anatomical single bundle ACLR is defined as a functional restoration of the native ACL and the femoral tunnel position resembles the native ACL biomechanical characteristic when placed posteriorly alongside the medial aspect of the lateral condyle over the anatomical footprint of the ACL anteromedial (AM) bundle, or between the AM and the posterolateral bundles, defined as central position [26]. There is evidence in the literature that the AM placement of the femoral tunnel during single bundle ACLR promotes good anteroposterior stability [13, 15], lower graft tension [5, 9], and lower revision rates [22]. Therefore, various methods have been developed to estimate the position of the femoral tunnel intraoperatively. The most used in clinical practice are the ‘clock face’ method [34] and anatomical landmarks [8], but they have limitations and can lead to non‐anatomical tunnel placement during ACLR [25].

The posterior cartilage of the lateral femoral condyle (point C), described by Cury et al. [21], is a point with little anatomical variation and easy recognition. It can be used as an anatomical landmark to mimic the AM bundle, but there are no studies in the literature that have evaluated the accuracy of this technique in vivo for positioning the femoral tunnel drilling in an outside in manner targeting the AM bundle aperture.

The aim of this study was to analyse the accuracy of the femoral tunnel placement during ACLR using point C as an anatomical reference landmark. It was hypothesised that point C is an anatomical reference capable of accurately assisting the surgeon in positioning the femoral tunnel closer to the centre of the AM bundle.

## METHODS

This is a prospective study of a series of consecutive cases. IRB number 63270422.0.0000.5479. All patients that were included in the study filled out the informed consent form prior to the procedure.

### Inclusion and exclusion criteria

The study included patients of both sexes, aged between 18 and 55 years, presenting with a unilateral, isolated ACL injury, who underwent surgical reconstruction performed by the same surgeon between December 2022 and December 2023. Eligible patients had no previous surgical procedures on the affected knee and an injury duration of more than 2 weeks but less than 5 years.

Patients with open growth plates or a coronal plane deformity exceeding 15° in varus or valgus requiring concomitant osteotomy were excluded. Additional exclusion criteria included the need for further ligamentous reconstructions (e.g., posterolateral or posteromedial corner, posterior cruciate ligament, or anterolateral and anteromedial ligaments), the presence of osteochondral lesions requiring procedures such as osteochondral transplantation or collagen membrane implantation, chondral degeneration graded higher than 2 according to the Kellgren and Lawrence classification (i.e., joint space narrowing > 5 mm compared to the contralateral knee with associated osteophyte formation) [16], systemic inflammatory diseases and a body mass index (BMI) greater than 35 kg/m².

Patients who met the study's inclusion criteria and provided informed consent underwent surgical intervention using Point C as a reference for the femoral tunnel placement, followed by postoperative computed tomography of the operative knee. In contrast, those patients who met the exclusion criteria did not undergo postoperative computed tomography.

### Surgical technique

**Figure 1:**
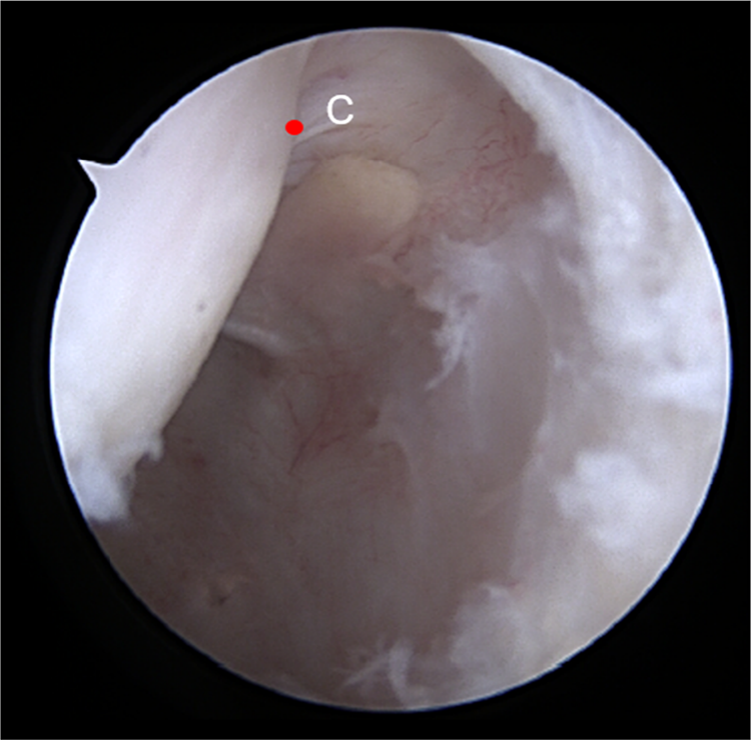
Arthroscopic visualisation of the right knee flexed at 90°, through the anteromedial portal from the most proximal and posterior point of the posterior cartilage of the lateral femoral condyle ‐ 'Point C' (red point).

**Figure 2:**
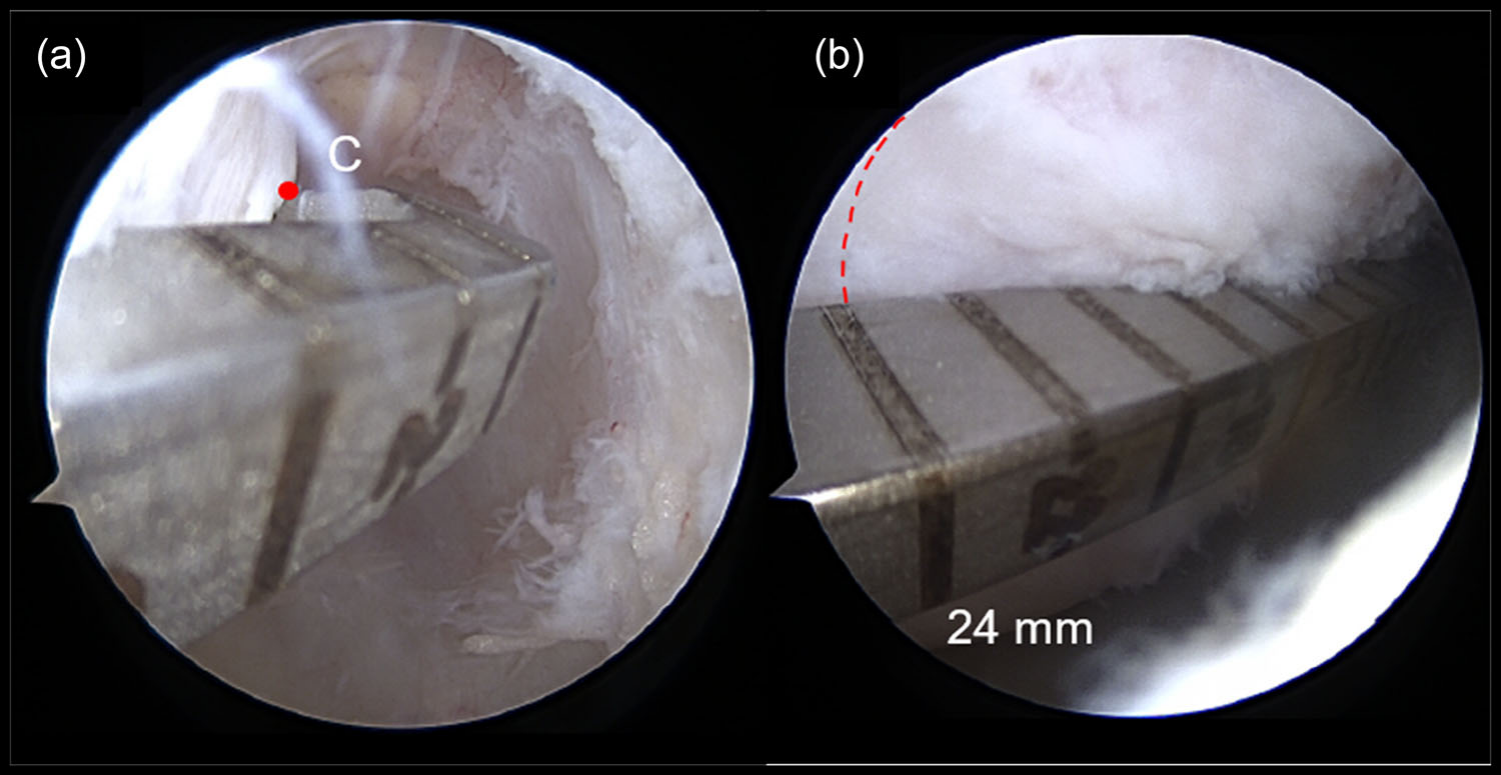
(a) Arthroscopic view of the right knee through the anteromedial portal showing the insertion of the femoral guide through the anterolateral portal, positioned parallel to the tibia, with the knee at 90°, with the posterior portion of the guide over Point C (red point). (b) Measurement is taken to the anterior margin of the lateral femoral condyle ‐ distance XY, measuring 24 mm in this patient (red dashed line).

The patients were positioned in a horizontal supine position under spinal anaesthesia [11]. The tendons of the gracilis and semitendinosus muscles ipsilateral to the injury were harvested to form a quadruple graft. Anteromedial, anterolateral, and accessory medial arthroscopic portals were created. After inspecting the joint cavity, evaluating meniscal and chondral injuries, the fibres of the ruptured native ACL were debrided and the medial wall of the lateral condyle was prepared using an arthroscopic shaver and radioablation (Coolcut®, Arthrex, Inc., Naples, Florida), identifying the posterior cartilage of the lateral femoral condyle (Point C) (Figure 1). With the knee flexed at 90°, the arthroscope was positioned through the accessory medial portal to allow better visualisation of the lateral intercondylar wall. A femoral guide, using the outside‐in technique (Curved Marking Hook Femoral ACL®, Arthrex, Inc., Naples, Florida), was inserted through the anterolateral portal, measuring from ‘Point C’ to the anterior edge of the femoral condyle, with the knee flexed at 90°, generating the XY distance (Figure 2a,b).

**Figure 3:**
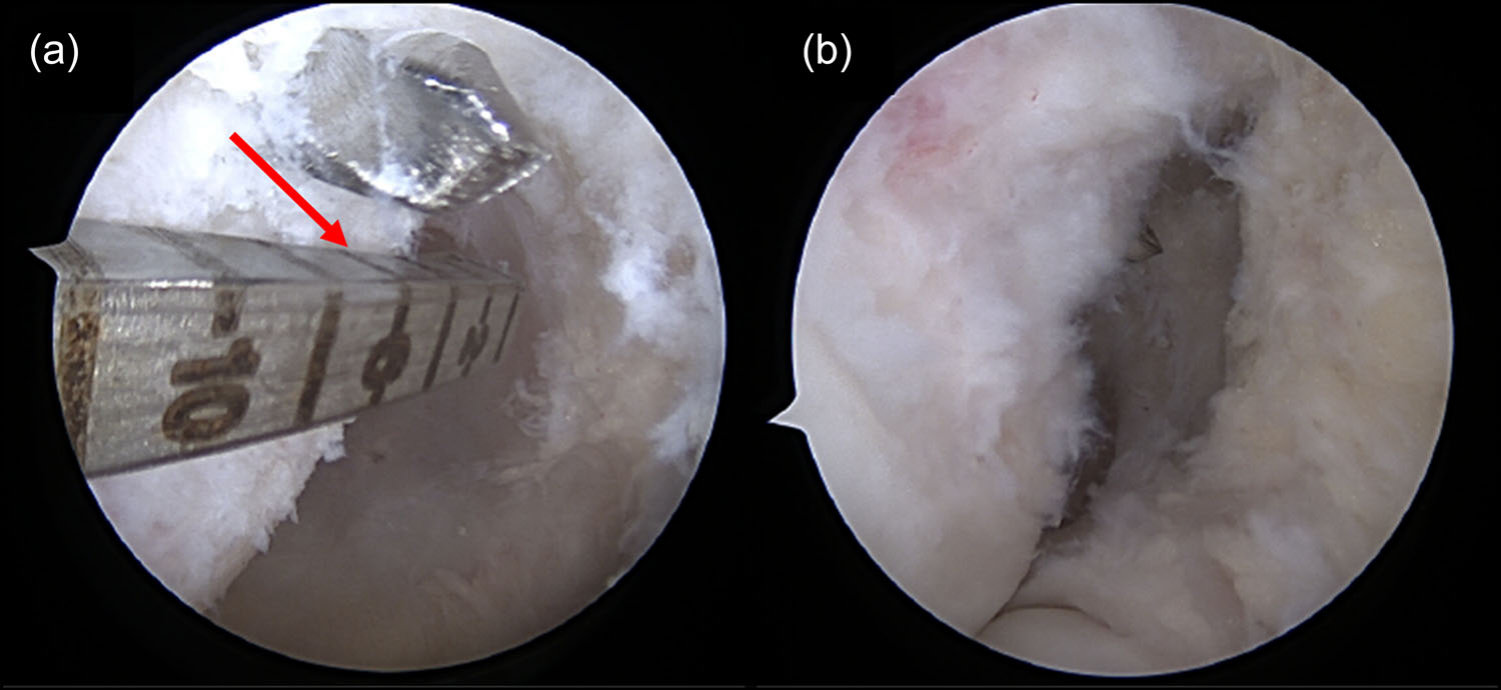
(a) Arthroscopic view of the right knee flexed at 90°. The arthroscope is positioned through the accessory medial portal. Through the anterolateral portal the guide wire is passed at 35% of the measurement of the lateral femoral condyle. In this patient, it measured 8 mm (red arrow) and 2 mm proximally. (b) When the view is switched to the anterolateral portal, the proximal positioning of the guide wire is observed.

**Figure 4:**
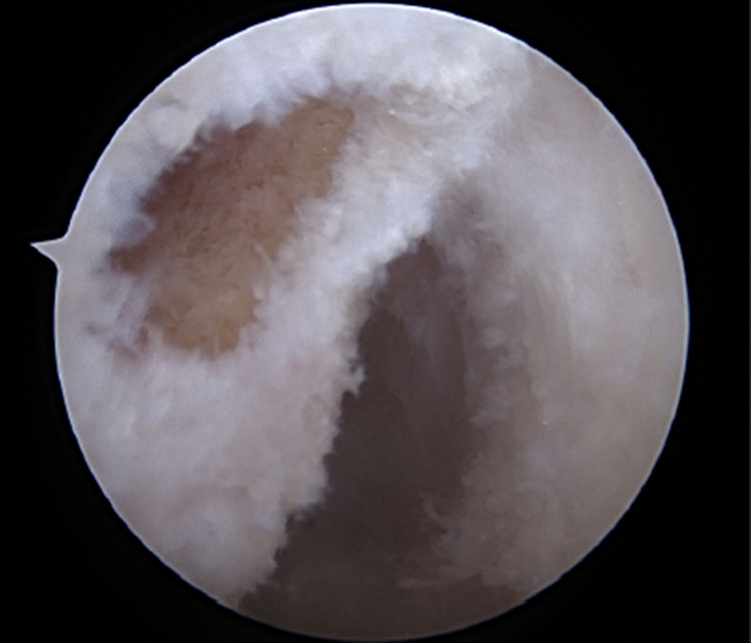
Relationship of the femoral tunnel over the anteromedial bundle, with the posterior portion of the lateral femoral condyle.

From the posterior limit of the XY distance, with the knee flexed at 90°, a value equivalent to 35% of XY was measured, from posterior to anterior, which represents the sagittal coordinate. From this coordinate, a Point 2 mm proximal, was marked with radiofrequency or ice pick through the accessory anteromedial portal, using as a parameter the ice pick tip, identifying the centre of the native AM bundle of the ACL. This measurement was described by Cury et al. [21], and differs from Bernard's quadrant. The arthroscopic measurement described by Cury et al. [21] was based on determining the axis of the femoral diaphysis, and a parallel line was drawn passing through the most proximal portion of the cartilage of the lateral femoral condyle (Point C) to the distal portion of the lateral femoral condyle cartilage. In contrast, the Bernard's quadrant was defined horizontally by the sagittal diameter of the lateral femoral condyle along Blumensaat's line, and vertically by a line perpendicular to Blumensaat's line, calculated as a percentage of the total height of the intercondylar notch. From this coordinate, the guide was used to pass the guide wire (Figure 3a,b), and finally, the femoral tunnel was created with a diameter based on the graft (Figure 4). The centre of the tibial tunnel was determined using the centre of the tibial footprint as reference [30] and drilling in an outside in manner with a specific guide. The hamstring graft was then moved between the tunnels, under arthroscopic visualisation, and fixed with bioabsorbable interference screws (Biocomposite, Arthrex, Inc., Naples, Florida), in an outside in manner, positioned superior to the graft in the femoral tunnel with the knee flexed at 90°, and anteriorly in the tibial tunnel with the knee in full extension under appropriate tension [11].

### Tomographic evaluation

**Figure 5:**
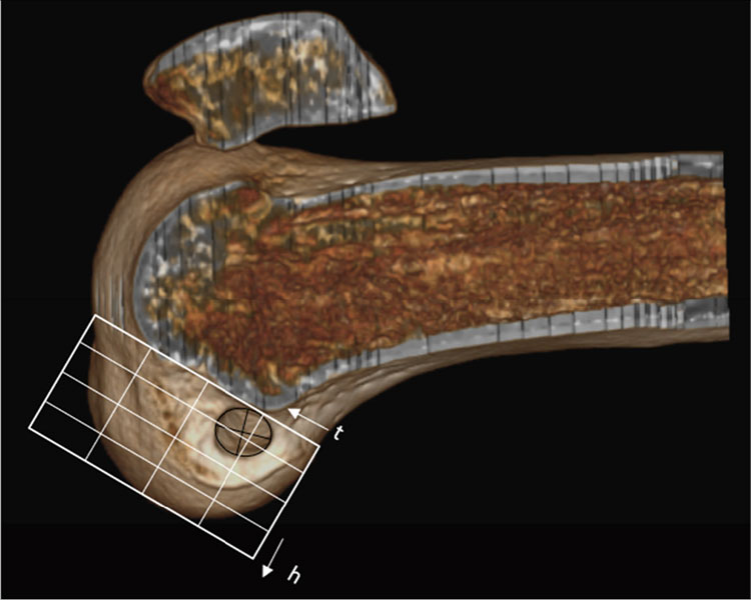
Evaluation with three‐dimensional computed tomography (CT)‐scanner with the medial femoral condyle removed and the knee in true sagittal view. A rectangular outline is created based on the Bernard quadrant method modified for 3D CT‐scanner. The horizontal limit corresponds to the total sagittal diameter of the lateral condyle along the Blumensaat line (distance *t*), measured as a percentage (0%–100%, deep/shallow). The vertical portion of the quadrant corresponds to the total distance from the intercondylar notch perpendicular to the Blumensaat line (distance *h*), also measured as a percentage (0%–100%, high/low). The location of the femoral tunnel within the quadrants is highlighted.

Within the first 24 h after the procedure, all patients that were included in the study underwent a multislice computed tomography (CT‐scanner) with 64‐channel technology (Somaton, Siemens Medical Solutions, Forchheim, Germany), with slice thickness of 1.25 mm. After the examination, the file was transferred to a three‐dimensional evaluation software (RadiAnt DICOM Viewer 64‐bit, Medixant, Poznan, Poland). The image was oriented to demonstrate a sagittal section in which a true side view of the knee is highlighted, and the medial condyle is removed at the centre of the intercondylar notch. Using Adobe Photoshop CS3 (Adobe Systems, San Jose, CA), a rectangular outline was created and the Bernard quadrant method modified for CT‐scan 3D reconstruction was used [14]. The horizontal axis of the quadrant method represents the total sagittal diameter of the lateral femoral condyle along Blumensaat's line (distance *t*), expressed as a percentage ranging from 0% to 100% (deep to shallow). The vertical axis corresponds to the perpendicular distance from the intercondylar notch to the Blumensaat's line (distance *h*), also expressed as a percentage from 0% to 100% (high to low). The position of the centre of the femoral tunnel was defined by the inner margin of the tunnel aperture within the intra‐articular space and was plotted within the quadrant grid. The horizontal (*X*) and vertical (*Y*) coordinates of this point were recorded as percentage values (Figure 5). The mean values of these coordinates were compared to the previously established anatomical reference for the AM bundle, based on the quadrant method described by Bernard et al. [2]. According to the systematic review by Xu et al. [33], the mean reported value for coordinate *X* is 24.2% ± 4.0%, and for coordinate *Y* is 21.6% ± 5.2%.

The CT‐scanner analysis, to evaluate the percentage coordinates of the positioning of each femoral tunnel, was performed by two surgeons with different levels of experience, one of whom has been a knee surgeon for over 30 years and the other a newly specialised knee surgeon. The two analyses were conducted at different times, with a 15‐day interval between them, and each evaluator did not have access to the evaluation performed by the other. Following sample collection and subsequent analyses, the mean measurement values were calculated and compared with the previously reported descriptions of the AM bundle footprint (horizontal coordinate = 24.2 ± 4.0% and vertical coordinate = 21.6 ± 5.2% [33]).

### Statistical analysis

A statistical significance level of 0.05 was set for this analysis. The distribution of demographic factors and imaging examination was characterised, with height and depth measurements considered normal within the reported measurement range, and values above or below the reference limits considered abnormal. To determine significance between the responses for the calculated prevalences, a Z‐test for two proportions was used. To measure the reliability of the variables, data from evaluator 1 were quantitatively analysed to calculate the intraclass correlation coefficient (ICC), standard error of measurement (SEE) and standard error of measurement (SEM). For the ICC, values ranging from 0 to 0.5 were considered poor, 0.5 to 0.75 were considered moderate, 0.75 to 0.9 were considered good, and 0.9 to 1 were considered excellent [18].

An a priori power analysis (GPower 3.1.9.4 software) with an expected high correlation (*r* = 0.5) from a previous study [29] determined the sample size of 45 with power of 0.80.

## RESULTS

**Figure 6:**
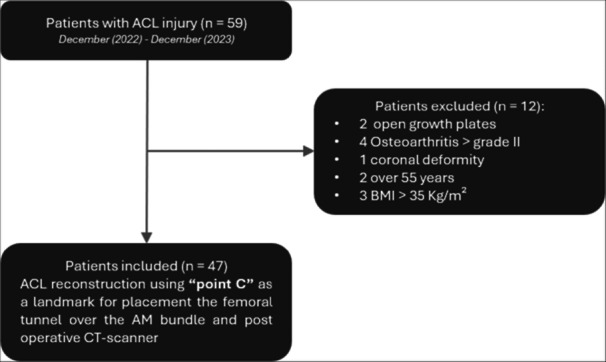
Flow chart describing patient selection. ACL, anterior cruciate ligament; AM, anteromedial; BMI, body mass index; CT‐scanner, computed tomography; *n*, number of patients.

Initially, 59 patients were included in the study. However, two had open growth plates and under 18 years old, two were over 55 years old, four presented with osteoarthritis greater than grade II according to Kellgren and Lawrence classification, one required a concomitant osteotomy due to coronal plane axis deviation and three had BMI greater than 35 kg/m². These patients were excluded from the analysis and didn't undergo CT‐scanner analyses. After the exclusion, 47 patients underwent ACLR using Point C as a parameter to mimic the AM bundle (Figure 6). No CT‐scanner was excluded due to inadequate imaging.

The average patient's age was 32 years, with a minimum age of 18 years and a maximum of 51 years. There were 28 right knees and 19 left knees. There were 14 female patients and 33 male patients. All patients had chronic ACL injury (more than 3 months of injury) (Table 1). The average distance from Point C to the anterior portion of the lateral femoral condyle was 23.3 mm, with the greatest distance being 26.0 mm and the smallest being 22.0 mm. The mean correlation value of Point C with the AM bundle in the horizontal coordinate intraoperatively was 7.7 mm, with the highest value being 9.0 mm and the lowest being 7.0 mm.

**Table 1:** Demographic data of the study.

Patient characteristics	Mean (range)	Percentage	Standard deviation	*N*
Age (Years)	32 (18–49)		10.1	47
Operative Knee				
Left		40.4%		19
Right		59.6%		28
Gender				
Male		70.2%		33
Female		29.8%		14
Body Mass Index (BMI) (kg/m²)	25.2 (20.1–30.4)		2.3	47
Tunnel diameter size^a^ (mm)	8.3 (7–10)		0.9	47
Time from injury to surgery (months)	8 (4–24)		4.7	47

Abbreviations: mm, millimetres; *N*, number of patients.

^a^ Measurement of the femoral diameter tunnel after the drilling.

**Figure 7:**
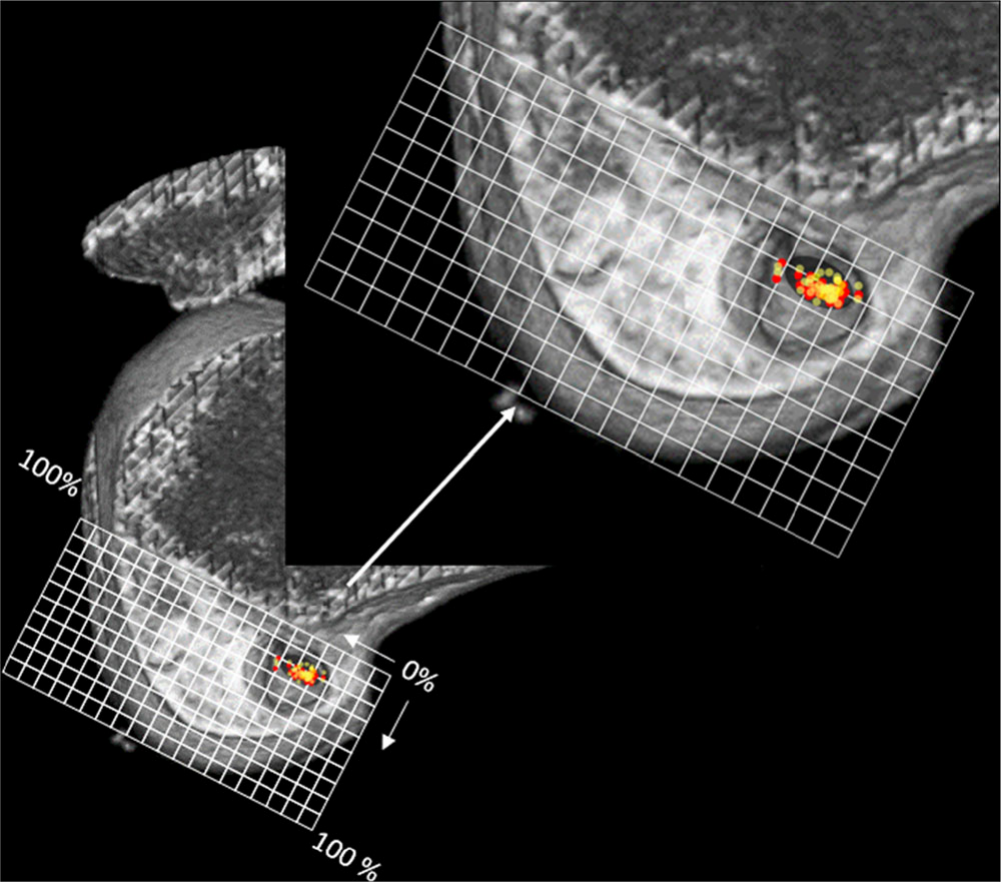
Representation of the Bernard quadrants with the representation of the horizontal (*t*) and vertical (*h*) coordinates in percentage. Highlighted in the upper image, the black circle represents the values of the coordinates of the anteromedial bundle in the literature (*t*: 24.2 ± 4.0% and *h*: 21.6 ± 5.2%). The 47 cases evaluated by Evaluator 1 in the first stage are represented in red. The 47 cases evaluated by Evaluator 2 are represented in yellow.

The average depth values (X coordinate) from evaluator 1 at Time 1 were 23.6%, and at Time 2 were 23.6%. The average height values (Y coordinate) at Time 1 were 22.7%, and at Time 2 were 22.0%. The analysis by the second evaluator at the third time had an average X coordinate of 23.6% and Y coordinate of 22.3% (Table 2) (Figure 7).

**Table 2:** Complete descriptive of quantitative factors.

	Mean	Median	Standard deviation	*N*	CI	SEE	ICC	SEM
Age (years)	32	33	10.1	47	2.9	1.5		
Distance CD (mm)	23.3	24	1.3	47	0.4	0.2		
Distance from Point C (mm)	7.7	8	0.7	47	0.2	0.10		
Depth 1st time evaluator 1	23.6%	23.6%	2.3%	47	0.7%	0.3%	0.942	0.6%
Depth 2nd time evaluator 1	23.6%	23.0%	2.6%	47	0.7%	0.4%		
Height 1st time evaluator 1	22.7%	22.4%	2.0%	47	0.6%	0.3%	0.791	0.9%
Height 2nd time evaluator 1	22.1%	22.1%	2.1%	47	0.6%	0.3%		
Depth 2nd evaluator	23.6%	23.0%	2.5%	47	0.7%	0.4%		
Height 2nd evaluator	22.3%	22.1%	1.8%	47	0.5%	0.3%		

Abreviations: CD, intraoperative evaluation of the distance from Point C to the anterior portion of the lateral femoral condyle; CI, confidence interval; ICC, intraclass correlation coefficient; mm, millimetres; *N*, number of patients; SEE, standard error of the estimate; SEM, standard error of measurement.

Out of the 47 patients evaluated, in all analyses, the Y distance (height) was within the margin of the AM bundle (between 21.6 ± 5.2% [33]). Regarding the X distance (depth), in Evaluator 1, three patients had a percentage outside the margin of the AM bundle (24.2 ± 4.0% [33]), with one located posteriorly (19.5% in Analysis 1 and 18.9% in Analysis 2 respectively), while two were performed anteriorly (29.5% and 28.9% in Analysis 1, and 31.3% in Analysis 2). In the third evaluation, performed by the second evaluator, two tunnels were performed posteriorly (19.4% and 18.2%), and two tunnels were performed anteriorly (31.2% and 28.9%) (Table 3). The accuracy for height was 100% in all three measurements. In depth, values ranged from 91.5% to 93.6% (Table 4). The intraclass correlation coefficient (ICC) values for intra and interobserver evaluation in depth and high were 0.942 and 0.791, respectively (Table 2).

**Table 3:** Distribution of qualitative factors.

	*N*	%	*p*‐value
Operative knee			
Right	28	59.6%	0.063
Left	19	40.4%	
Gender			
Female	14	29.8%	<0.001
Male	33	70.2%	
Height (T1 ‐ Evaluator 1)			
Normal	47	100%	<0.001
Altered	0	0%	
Height (T1 ‐ Evaluator 2)			
Normal	47	100%	<0.001
Altered	0	0%	
Height (T2 ‐ Evaluator 1)			
Normal	47	100%	<0.001
Altered	0	0%	
Depth (T1 ‐ Evaluator 1)			
Normal	44	93.6%	<0.001
Altered	3	6.4%	
Depth (T1 ‐ Evaluator 2)			
Normal	43	91.5%	<0.001
Altered	4	8.5%	
Depth (T2 ‐ Evaluator 1)			
Normal	44	93.6%	<0.001
Altered	3	6.4%	

Abbreviations: N, number of patients; T1, Time 1; T2, Time 2.

**Table 4:** Accuracy of Point C.

	Height		Depth	
	Accuracy	CI	Depth	CI
T1 ‐ Evaluator 1	100%	0%	93.6%	7.0%
T1 ‐ Evaluator 2	100%	0%	91.5%	8.0%
T2 ‐ Evaluator 1	100%	0%	93.6%	7.0%

Abbreviations: CI, confidence interval; T1, Time 1; T2, Time 2.

## DISCUSSION

The most important finding of this study was the accuracy in using point C to create the femoral aperture in the AM bundle during single bundle ACLR with outside in technique. The technique showed good accuracy, with the tunnel being created within the literature values of the AM bundle in 100% of cases in terms of height. In terms of depth, 93.6% of patients had the centre of the tunnel within the coordinates of the AM bundle according to Evaluator 1, and 91.5% according to Evaluator 2. These results are in line with the study by Cury et al. [21], since the depth parameter showed greater variability between studied specimens, therefore this lower accuracy in the depth parameter was already expected.

The depth results in percentage of the AM bundle in this study were 23.6% ± 2.3 and in height were 22.7% ± 2.0. These data of AM bundle within the Bernard quadrant system were similar to the systematic review by Xu et al. [33]. Tsukada et al. [28] showed that the AM bundle within the quadrant system was described as 25.9% ± 2.0 from the posterior lateral femoral cartilage to the anterior wedge of the femoral cartilage, and 17.8% ± 2.9 from the Blumensaat's line to the inferior femoral cartilage wedge. There are others systematic reviews that aim to determine the femoral ACL footprint within the quadrant system. Piefer et al. [24] showed that the centre of the AM bundle was described as 29.5% of the proximal‐to‐distal length of the lateral femoral intercondylar notch wall, and 2.5 mm anterior to the posterior cartilage margin. These discrepancies across studies may be explained by different methods of dissection, the variety of imaging modalities and the difficulties determining the ACL femoral footprint. Moreover, while the original Bernard quadrant method was developed for radiographic assessment, the present study employed a modified version adapted for CT imaging. Despite the variability, this paper's results were similar to the findings of Xu et al. [33] and Tsukada et al. [28].

Several surgical techniques have been developed to assist the surgeon in creating the femoral tunnel in ACLR. The most used are the clock face technique [34], anatomical landmarks [8], and the native ACL remnant [7]. The clock face technique has several limitations, such as not evaluating the depth of the intercondylar roof and not considering the anatomical landmarks of the ACL [31]. On the other hand, the native ACL remnant and anatomical landmarks, such as the lateral intercondylar ridge and bifurcate, may not be present in chronic injuries and are not always easily identified [7].

Considering the great variability of these anatomical landmarks, some studies have sought to identify new anatomical references to facilitate the identification of the femoral tunnel. Weiler et al. [29] used the posterior horn of the lateral meniscus to create the femoral tunnel in the central position drilling the tunnel through the AM portal. The use of the posterior portion of the lateral femoral condyle cartilage as an anatomical landmark for creating the femoral tunnel has been previously described in the literature as a reliable landmark for positioning the femoral tunnel during ACLR [6, 35]. Dong et al. [6] in a retrospective case series, used the most proximal and posterior portion of the lateral femoral cartilage as an anatomical landmark, employing an arthroscopic ruler positioned 12.0 mm shallow to the cartilage and 3.0 mm high, utilising the inside‐out drilling technique. They identified proper positioning and good postoperative outcomes. Comparing this study with our analysis, the distance of the femoral tunnel in our assessment showed variation, ranging from 9.0 to 7.0 mm. We attribute this to anatomical differences in patient size relative to their height. According to Shi et al. [27] the distance from the most proximal and distal portion of the lateral femoral cartilage was 9.0 ± 1.2 mm. To the best of our knowledge, no previous study has described a technique—along with its in vivo reproducibility—using this anatomical landmark as a reference landmark to replicate the AM bundle in single‐bundle, outside‐in ACLR.

Regarding the positioning of the femoral tunnel on the AM footprint or at the central point, biomechanical analyses have shown non‐isometric behaviour when the femoral tunnel is in the central portion [15], and eccentric positioning near the AM bundle may be associated with better clinical outcomes [19]. In cadaveric studies, positioning of the femoral tunnel near the AM bundle has demonstrated better control of both anteroposterior and rotatory stability compared to positioning near the posterolateral bundle [17]. Similarly, ACLR with the femoral tunnel positioned near the AM bundle has shown less tension on the graft [3]. In prospective studies, the closer the femoral tunnel is to the origin of the AM bundle, compared to a tunnel created in the central portion, the better the postoperative clinical outcomes with objective evaluation using the Lachman test and International Knee Documentation Committee (IKDC) score [19], and lower revision rates [22]. In the present study, our objective was not to compare the two surgical techniques, but rather to evaluate the accuracy of the surgical technique using Point C as an anatomical reference to create the femoral tunnel as close as possible to the AM bundle. A biomechanical and functional postoperative analysis would be necessary to compare the central and AM tunnels, which was not proposed in this study.

Some anatomical studies have shown variability in size and shape of the femoral insertion of the ACL [7, 23], making the anatomical reconstruction of the femoral tunnel complex. Another factor that influences femoral tunnel creation is the morphology of the intercondylar roof, which significantly affects the tunnel positioning and may lead the surgeon to create a more distal tunnel in cases of excessive inclination of the intercondylar roof [12]. This anatomical variability was not considered in the study, but it may be related to cases where the depth did not meet the parameters adopted for the AM bundle (three cases according to Evaluator 1 and four cases according to Evaluator 2). Further studies are needed to correlate the depth of the femoral tunnel with the morphology of the intercondylar roof.

Based on the results of this study, Point C can be considered a reliable anatomical landmark for creating the femoral tunnel mimicking the AM bundle during ACLR. The authors believe that surgeons should use multiple intraoperative parameters to create the tunnel as anatomically as possible and Point C can be used as one of these parameters.

The study has some limitations. First, it was based solely on imaging assessment. There is a lack of long‐term clinical evaluation assessing functional outcomes, satisfaction levels, and complications. More prospective analyses are needed. Second, for the analysis of the technique's accuracy in this study, only one surgeon performed the surgeries. However, to clarify the external validation of the technique, we believe that the procedure should be performed by more surgeons to evaluate future results in studies with a larger sample size. Third, the femoral tunnel in this technique was created using the outside‐in approach, no cases were performed drilling the femoral tunnel through the anteromedial portal. Also, the study is a case series investigation based on short‐term follow up without control group. Although this paper's objective was to evaluate the tunnel position during single bundle ACLR, the fact that this is a consecutive case series is a limitation to inferring the clinical outcomes of the technique.

## CONCLUSION

Point C has been shown to be an anatomical landmark with good accuracy for creating the femoral tunnel in the AM bundle during ACLR with outside in technique.

## AUTHOR CONTRIBUTIONS

Viktor Nelson Mazzola Corrêa performed the surgical procedure on the patients and data collection. Nayra Deise dos Anjos Rabelo and Ricardo de Paula Leite Cury were responsible for the analysis and interpretation of the data. Viktor Nelson Mazzola Corrêa, Alfredo dos Santos Netto, and Nayra Deise dos Anjos Rabelo were responsible for preparing the manuscript. Luiz Gabriel Betni Guglielmetti, Victor Marques de Oliveira, and Ricardo de Paula Leite Cury were responsible for the review and critical analysis of the article. Ricardo de Paula Leite Cury provided final approval of the version for publication and agreed to be responsible for all aspects of the work, including issues related to the accuracy and integrity of any part of the work.

## CONFLICT OF INTEREST STATEMENT

The authors declare no conflict of interest.

## ETHICS STATEMENT

The study was approved by the Ethics and Research Committee of the Irmandade da Santa Casa de Misericórdia de São Paulo. Approval number CAAE 63270422.0.0000.5479. All patients filled out the informed consent form prior to the procedure.

## Data Availability

The data that support the findings of this study are available from the corresponding author upon reasonable request.
